# Storage Potential of Cactus Mucilage Powder for Incorporation into Foods and Production of Biopolymeric Films

**DOI:** 10.1021/acsomega.4c05691

**Published:** 2024-10-14

**Authors:** Jheizon Feitoza do Nascimento Souza, Lucas Vinícius Pierre de Andrada, Lady Daiane Costa de Sousa Martins, Andréa Monteiro Santana Silva Brito, Lúcio José Vieira Silva, Ivo Diego de Lima Silva, Glória Maria Vinhas, Thieres George Freire da Silva, Adriano do Nascimento Simões

**Affiliations:** † Academic Unit of Serra Talhada, Rural Federal University of Pernambuco, Serra Talhada, Pernambuco 56903465, Brazil; ‡ Biosciences Institute, Botucatu Campus, São Paulo State University “Julio de Mesquita Filho”, Botucatu, São Paulo 18618689, Brazil; § Department of Chemical and Engineering, Federal University of Pernambuco, Recife, Pernambuco 50711340, Brazil; ∥ Federal Rural University of the Semi-arid, Mossoro, Rio Grande do Norte 59625900, Brazil

## Abstract

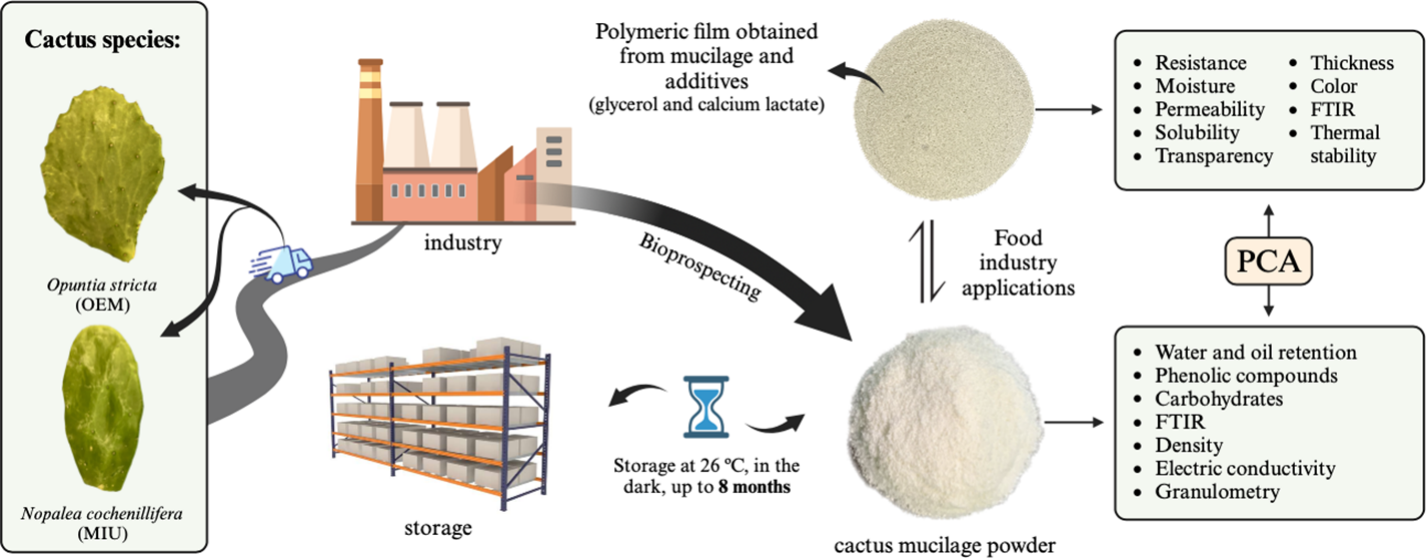

The objective was to investigate the physicochemical stability of stored cactus pear mucilage and assess the technological feasibility to produce polymeric films. Mucilage of cactus pear species *Nopalea cochenillifera* (L.) Salm-Dyck MIU and *Opuntia stricta* (Haw.) Haw—OEM was extracted and stored for 2, 4, 6, 8, and 10 months in the absence of light at a temperature of 26.5 °C and relative humidity of 41.3%. At each storage time, polymeric films were produced using hydrated mucilage (4%, weight—w/volume—(v)), glycerol (60%, v/v), and calcium lactate (2%, w/v). Among the species, MIU stood out due to its higher water and oil retention, but it also presented higher levels of phenolic compounds, and more intense peaks in Fourier transform infrared spectrophotometry (FTIR) analysis. On the other hand, OEM is richer in carbohydrates, denser, and electrically conductive. The characteristics highlighted for each species are also observed in the principal component analysis (PCA). Both species are equally soluble in water, and more than 60% of their granules have a diameter of 250 mm. The resulting films of MIU exhibited increased resistance and permeability but were less soluble and transparent. Microscopically, greater homogeneity was observed, and the films were thicker, whitish, and thermally stable. Both species have the potential for producing polymeric films with various applications in the food industry, particularly as edible coatings.

## Introduction

The development of new biomaterials from agricultural byproducts and residues is a global trend and one of the challenges of the new millennium, aiming to promote environmental protection through green chemistry and innovative design, in addition to the use of natural, eco-friendly products. Mucilage is an interesting biopolymer that has been applied in the food, cosmetics, and pharmaceutical industries^1^ that guarantees the maintenance of the organoleptic characteristics of foods for a longer time. Studies that assess the durability of large-scale mucilage extractions when stored for the production of biomaterials are insufficient. Yet given the characteristics of the raw material, its production, and its different availability throughout Brazil, it is important to examine the question of storage.

The elastic characteristic of mucilage and its ability to form a molecular network makes it applicable for food packaging as edible polymeric coatings, increasing its shelf life and attributing value to the product.^2^ The great challenge for the elaboration of biopolymeric films using cactus pear are environmental conditions,^3^ management,^4^ and genetic variability, since different species result in mucilages with different properties and, consequently, in different biopolymeric films. Tests with powdered mucilage have been carried out with the freshly extracted material,^3,5^ but the viability of storage is still unknown. This information is essential to industry for the identification of the ideal storage period needed to maintain the filmogenic properties of the mucilage and for the formulation of biopolymeric films based on each genus or even on each clone.

To ensure the effectiveness of the films, it is necessary to characterize the mucilage by evaluating fundamental parameters such as carbohydrate content, which is an abundant component of the polymeric matrix;^6^ electrical conductivity, closely linked to viscosity and rheological properties when used as coating bases;^7^ aspects of hydrophilicity and hydrophobicity,^8^ important guidelines regarding material use and its interaction when on food surfaces; and finally, the potential for film formation, a key indicator of the coating or film’s ability to create an effective barrier against external agents. The validation of these parameters ensures the quality and functionality of the mucilage when stored.

Considering that industry can store the mucilage to use it as a raw material for food applications or in the production of films and coatings, it becomes imperative to study the long-term storage potential of mucilage to assess its physicochemical and technological stability. Therefore, the aim of this study was to evaluate the physicochemical stability of stored mucilage from two cactus species and the quality of the biopolymeric films.

## Material and Methods

### Mucilage Extraction and Characterization of Its Optical, Physical–Chemical, and Technological Properties

Cladodes were collected at the International Reference Center for Agrometeorological Studies of Cacti and Other Forage Plants, in the municipality of Serra Talhada, PE (7°59 ′S; 38°15 ′W and 431 m). According to the Köppen classification system, the climate in the region is BShw type.^9^ The average annual precipitation is 642 mm, the average air temperature is 24.8 °C, the relative humidity (RH) is 62% and the atmospheric demand for water is greater than 1800 mm per year.^10^

The mucilage was obtained according to Gheribi et al.,^3^ with some modifications. Cactus pear cladodes, *Nopalea cochenillifera* (L.) Salm-Dyck MIU (100004) and *Opuntia stricta* (Haw.) Haw—OEM (200016), with an average size of 100–230 mm, obtained from the middle third of the plant, were harvested, weighed, washed in running water and the epidermis was removed. The remaining aquiferous parenchyma was ground in a multiprocessor (Philips Walita, ri7775, Barueri, Brazil) with ethyl alcohol (99.8% PA) in a 2:3 ratio (vegetable material/alcohol) and homogenized. Successive washings with ethanol were carried out to remove the remaining chlorophylls and obtain a precipitate that was as whitish as possible. The precipitate was dried in an oven at 55 °C for 48 h. After that, the dry mucilage was pulverized using a micro mill (Tecnal, Type Willye, TE-648), obtaining a whitish powder. When necessary, part of the powder obtained was hydrated at a concentration of 4% w/v (4000 g of powder to 100 mL of distilled water) for mucilage analyses. The mucilage obtained was divided and analyzed right after extraction (month 0), and at intervals of 2 months until 10 months were completed (2, 4, 6, 8, and 10 months). Storage was carried out in the absence of light, at an average temperature of 26.5 °C and relative humidity (RH) of 41.3%.

### Experimental Design and Statistical Analysis

The experiments were conducted in a completely randomized design (DIC) with three replications. The analyses were carried out in triplicate. The data were submitted to normality tests and Tukey’s test at 5% probability with the aid of the R software version 4.2.1. Graphs were prepared using SigmaPlot software, version 14. For principal component analysis (PCA), the R software tool (R CORE TEAM, 2022) was used, in which the data means of the properties studied were decomposed into sets of orthogonal vectors. The results of the correlation matrix were displayed in biplots with their distribution in the space of ordinations, variances, and Pearson’s correlation. Graphs were created using SigmaPlot software, version 14 (Systat Software Inc., 2020) and OriginLab version 8.5.

### Agro-Industrial Yield

The agro-industrial yield was calculated by using the following formula:
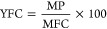
where YFC = yield of fresh cladode, %; MP = mass of powdered mucilage, g; and MFC = mass of fresh cladode, g.

### Total Titratable Acidity, Electrical Conductivity, Density, Carbohydrates, and Phenolic Compounds

The total titratable acidity was determined according to Astello-García et al.,^11^ with some modifications; using 0.1 N aqueous hydroxide (NaOH) solution and equivalent gram of citric acid (64.02). A 1% phenolphthalein solution was used. The results were calculated by the following formula and expressed in % citric acid.
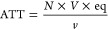
where ATT = total titratable acidity (% citric acid); *N* = normality of the sodium hydroxide solution (0.1 N); *V* = volume of the NaOH sample used in the titration (mL); eq = gram equivalent of citric acid (64.02); and *v* = volume of sample used (mL).

Electrical conductivity was performed using a conductivity meter (TECNAL, Tec-4MP, Piracicaba, Brazil). The sensor was inserted directly into the hydrated mucilage samples, and the reading was made. The results were expressed in mS/cm.

The density was obtained by using a glass pycnometer with a thermometer. 1 g of mucilage was weighed on an analytical balance (BIOPRECISA, FA2104N, Curitiba, Brazil), and the mucilage was slightly hydrated in order to avoid invalid results and then inserted into the pycnometer. The pycnometer was filled with water, and the system was closed with the thermometer. The system (pycnometer + thermometer + sample + water) was weighed, and the temperature was measured. The system (pycnometer, thermometer, and water) necessary for the calculation was also weighed. The results were calculated using the following formula and expressed in g/mL.
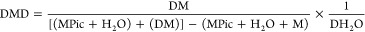
where DMD = dry mucilage density (g/mL); DM = dry mucilage mass (g); MPic = mass of the pycnometer with thermometer (g); H_2_O = water; M = mucilage; and DH_2_O = absolute density of water as a function of temperature.

The soluble carbohydrate content was obtained according to the methodology described by Dubois et al.,^12^ with modifications. The hydrated mucilage (2 mL) was centrifuged (Hettich, MIKRO 220, Berlin, Germany) at 10,000 rpm and 4 °C for 21 min. A 10 μL aliquot of the sample’s crude extract was added to 490 μL of deionized water, 500 μL of 5% phenol, and 2.5 mL of 98.08% concentrated sulfuric acid and placed in test tubes and shaken. Subsequently, the tubes were left to rest for 10 min in a tray containing water at room temperature. After this time had elapsed, the readings were taken in a spectrophotometer (Model Libra S8, Biochrom, Cambridge, U.K.) at 490 nm. The blank consisted of 500 μL of deionized water, 500 μL of 5% phenol, and 2.5 mL of 98.08% concentrated sulfuric acid. The results were expressed in g/(100 g) of dry matter and quantified based on the equation obtained for the standard curve, whose reference carbohydrate was glucose.

Total phenolic compound content was determined according to Jaramillo-Flores et al.,^13^ with some modifications. The 2 mL volume of hydrated mucilage was placed in a centrifuge (Hettich, MIKRO 220, Berlin, Germany) at 10,000 rpm, at 4 °C for 21 min. A 250 μL aliquot of the supernatant was combined with 250 μL of the Folin Ciocalteu reagent (1 N). The mixture was homogenized in a vortex (TECNAL, AP56, Araraquara, Brazil) and allowed to rest for 2 min. Then, 500 μL of 20% sodium carbonate (w/v) was added, and the mixture remained at rest for another 10 min. Finally, the readings were performed on a spectrophotometer (Biochrom, Libra S8, Cambridge, England) at 757 nm. Total polyphenol content was expressed in g/(100 g) of dry matter.

### Granulometry (GRA)

The determination of the size of the mucilage grains was carried out using granulometric sieves (ASTM 35, 60, and 270). Samples of 5.000 g of mucilage were weighed using a semianalytical balance (OHAUS 4100/0.01 g) and passed through the sieves with manual agitation. What was retained in each of the meshes was weighed.

### Water and Oil Holding Capacity (OHC)

The water holding capacity (WHC) was estimated using the method described by de Andrade Vieira et al.,^8^ with modifications. Mucilage samples (0.2000 g) were added to 10 mL of distilled water in Falcon tubes, kept for 1 h at room temperature, and stirred for 5 s every 15 min. They were then centrifuged at 5000 rpm for 20 min. The supernatant was discarded, and the tube material was placed in an oven at 55 °C for 30 min to remove the remaining water. The WHC was expressed as the amount of water retained in mucilage weight (g), calculated by the equation below:
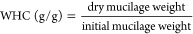
The oil holding capacity (OHC) was measured according to the methodology proposed by de Andrade Vieira et al.,^8^ with modifications. Samples of 0.1000 g of mucilage were added to 10 mL of soybean oil in Falcon tubes and shaken at 200 rpm in an incubator (TECNAL, model TE 420) for 1 h. The mixture was centrifuged at 5000 rpm for 15 min, the supernatant was discarded, and the precipitate was dried in an oven at 55 °C for 24 h. The OHC was calculated, and the results were expressed in grams of adsorbed oil per gram of mucilage, as follows:
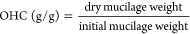


### Film Formulation and Study of Its Optical, Physical–Chemical, Mechanical, and Structural Properties

For the preparation of biopolymeric films, the methodology proposed by Brito et al.^14^ was followed, with modifications. The powder resulting from the extraction and stored for study for 10 months was also used for the elaboration of the biopolymeric films. These were also studied at intervals of 2 months until the 10th month. For this purpose, the mucilage was hydrated at a ratio of 4% (w/v) (4000 g of powder to 100 mL of distilled water) to form an emulsion. To this was added glycerol (plasticizer) and calcium lactate in standard concentrations of 60 and 2%, respectively. The emulsion was heated to 70 °C for 10 min. The material was dried in an oven at 55 °C for 24 h, and then the biopolymeric biofilm formed was removed and stored for analysis.

### Visual Appearance, Photomicrographs, and Color

Comparative and visual observations of the material were made with photographs taken on a smartphone (Apple iPhone XR). Photographs were also taken under an optical microscope with a 4× magnification lens. The microscopic structure of the film surface was analyzed through scanning electron microscopy (SEM). For this purpose, the samples were mounted on supports and coated with gold using a DENTON VACUUM metallizer, model DESK V. Subsequently, the samples were inserted into a scanning electron microscope TESCAN, model VEGA3, equipped with a tungsten filament. The images were captured under an acceleration voltage of 20.0 kV and at magnifications of 85 and 380×.

The color was obtained through a colorimeter (RS 232 with RGB serial output, 1002) with values obtained in the RGB system. The data obtained by the colorimeter were divided by 4 to suit the RGB scale (0–255) and then converted into the CIE color scale L*, a*, b*,^15^ where L* corresponds to variations in sample brightness (0–100, darkest to brightest), a* corresponds to variations from green (−*a*) to red (+*a*), and b* is attributed to variations from blue (−*b*) to yellow (+*b*). Value conversion was performed using online software available on a public Web site: http://www.easyrgb.com/en/convert.php#Result. Subsequently, the a* and b* data set was converted and expressed in Chroma saturation values (C*) according to the methodology of Espino-Díaz et al.,^16^ in which:
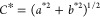


### Transparency, Thickness, Moisture Content (MC), Water Solubility, and Water Vapor Permeability

Transparency was determined using rectangular segments of the films placed in cuvettes of a spectrophotometer perpendicular to the path taken by the light to obtain the absorbance at 600 nm. An empty cuvette was used as a control. To obtain transparency, absorbance was converted to transmittance with the following formula:
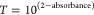
For determination of transparency. The transmittance was determined according to the formula:
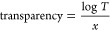
where transparency = % *T*/mm; *T* = transmittance (%); and *x* = film thickness (mm).

The thickness (mm) was measured at 10 random points on the films with a digital micrometer, with a resolution of 1 μm, and an average was performed.^3^

The moisture content was measured by cutting the films into 1.0 × 1.0 cm^2^ and weighing them. After this, they were put in the oven for 24 h at 55 °C until they reached constant weight (weight of the dry sample). The final weighing of the fragments determined the moisture content of the biopolymeric films, calculated by the formula:
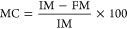
where MC = moisture content (%); IM = initial mass of fragments (g); and FM = final mass of fragments (g).

Water solubility was performed with 1.0 cm^2^ fragments of biopolymeric films, dried in an oven at 55 °C for 24 h, cooled to room temperature in a desiccator, weighed, and immersed in 12.5 mL of distilled water at 25 °C for 30 min. After that, the undissolved fragments were stored in the oven for 24 h at 55 °C, placed in the desiccator to cool, and weighed at the end of the process. The solubility in water was determined by the formula:
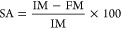
where SW = solubility in water (%); IM = initial mass of fragments (g); and FM = final mass of fragments (g).

Permeability was measured according to the methodology proposed by Sukhija et al.,^17^ with some modifications. Film samples were positioned to cover 20 mL polypropylene beakers containing about 15 g of calcium carbonate (CaCO_3_), with an approximately 10 mm distance between the carbonate and the sample. The beakers were then placed in a desiccator with the temperature and relative humidity monitored at 25 °C and 70% RH. Water vapor transport was determined by the weight gained in the cups; the slopes (weight changes as a function of time) were calculated by linear regression (*R*^2^ > 0.99). The water vapor permeability (g m/m^2^ day kPa) was calculated according to the formula:
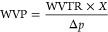
where WVTR = water vapor transmission rate (g/m^2^ day) defined as the slope (g/day) divided by the transfer area (m^2^); *X* = film thickness (m); and Δ*p* (kPa) = partial water vapor pressure difference across the film. (Δ*p* = *p*(RH_2_ – RH_1_) = 2.22 kPa, where *p* is the saturation vapor pressure of water at 25 °C, RH_2_ = 70% and RH_1_ = 0%).

### Tensile Strength (TS) and Degradation by Temperature

Tensile strength (TS) was performed using a tensile machine (IMPAC, IP-AEL-A-50, São Paulo, Brazil) according to the method proposed by Gheribi et al.,^3^ with modifications. For each film formulation, three rectangular film strips (20 mm × 70 mm) were tested at a speed of 100 mm/min using a double clamp with a separation of 50 mm.

The thermal stability of the films was evaluated by thermogravimetric analysis (TGA) using a TGA 2 thermobalance (Mettler Toledo). The experiment was carried out under a nitrogen atmosphere with heating sweeps from 35 to 600 °C and at a heating rate of 10 °C/min for each sample.

### Fourier Transform Infrared (FTIR) Spectrophotometry

Spectral analyses in the mid-infrared region were performed on a Fourier transform infrared (FTIR) spectrophotometer (Frontier by PerkinElmer) using the universal attenuated total reflection (UATR) accessory. The spectra were obtained in the region of 4000–400 cm^–1^, resolution 8 cm^–1^, and eight scans. Air was used for the blank. The measurements were performed directly on the mucilage-based polymer under a diamond crystal.

## Results

### Physical–Chemical Stability of the Mucilage

The total titratable mucilage acidity did not differ between the species studied; also, the acidity did not change after 10 months of storage (Table 1). On the other hand, the electrical conductivity and density of the OEM species showed significantly higher values compared to MIU (Table 1). During storage, electrical conductivity, density, total carbohydrates, and total phenolic compounds (TPC) reduced significantly, regardless of the species (Table 1). In addition, OEM had higher values of carbohydrates, while MIU had higher values of phenolic compounds (Table 1).

**Table 1:** Physical–Chemical Stability of the Mucilage and Functional Properties of the Polymeric Films of *N. cochenillifera* (L.) Salm-Dyck MIU and *O. stricta* (Haw.) Haw—OEM, Immediately after the Cladode Harvest and after 2, 4, 6, 8, and 10 months^a^^b^

			time (months)					
properties			start (0)	2	4	6	8	10
luminosity (L*)		MIU	65.20^Ca^	73.50^Ba^	70.57^Ba^	85.45^Aa^	87.81^Aa^	69.36^Ba^
		OEM	58.05^Cb^	72.72^Bb^	70.03^Bb^	82.34^Ab^	86.03^Ab^	69.89^Bb^
chroma (C*)		MIU	18.95^BCb^	19.24^BCa^	16.48^Cb^	21.20^BCa^	21.97^ABb^	26.63^Aa^
		OEM	27.65^Aa^	20.36^Ba^	20.26^Ba^	23.94^ABa^	25.90^Aa^	19.52^Bb^
total titratative acidity (% citric acid)		MIU	0.26^Aa^	0.26^Aa^	0.26^Aa^	0.26^Aa^	0.26^Aa^	0.26^Aa^
		OEM	0.26^Aa^	0.26^Aa^	0.26^Aa^	0.26^Aa^	0.26^Aa^	0.26^Aa^
total soluble carbohydrates (g/(100 g) DM)		MIU	20.1^Ab^	19.7^ABb^	17.4^ABCb^	19.0^ABb^	16.6^Cb^	19.0^BCb^
		OEM	30.6^Aa^	27.9^ABa^	28.1^ABCa^	27.7^ABa^	25.1^Ca^	24.4^BCa^
total phenolic compounds (g/(100 g) DM)		MIU	3.39^Aa^	3.17^Aa^	2.22^Ca^	1.78^Da^	1.81^Da^	2.71^Ba^
		OEM	3.16^Ab^	2.87^Bb^	2.05^Ca^	1.23^Db^	1.37^Db^	1.28^Db^
tensile strength (MPa)		MIU	3.41^Ba^	3.72^Aa^	2.69^Ea^	2.53^Fa^	3.03^Da^	3.20^Ca^
		OEM	1.20^Db^	1.88^Cb^	2.13^Ab^	2.04^ABb^	2.14^Ab^	2.0^BCb^
transparency (% *T*/mm)		MIU	5.21^Cb^	6.32^BCa^	5.81^Ca^	5.35^Cb^	7.08^Ba^	10.39^Aa^
		OEM	6.42^Aa^	7.09^Aa^	6.19^Aa^	6.87^Aa^	7.14^Aa^	7.17^Ab^
thickness (mm)		MIU	0.38^Aa^	0.34^Aa^	0.38^Aa^	0.34^Aa^	0.27^Ba^	0.23^Bb^
		OEM	0.32^ABb^	0.27^BCDb^	0.32^ABCb^	0.27^CDb^	0.25^Da^	0.36^Aa^
electrical conductivity (mS/cm)		MIU	978.4^Bb^	846.6^Db^	819.9^Eb^	910.4^Cb^	853.1^Db^	1365^Aa^
		OEM	1117^Ba^	1036^Da^	1076^Ca^	988.6^Ea^	1033^Da^	1346^Ab^
water retention capacity (g/g)		MIU	13.64^Aa^	12.93^BCa^	14.09^Aa^	12.79^Ca^	13.52^ABa^	6.74^Da^
		OEM	7.29^Ab^	7.15^Ab^	7.23^Ab^	7.01^Ab^	7.30^Ab^	4.57^Bb^
oil holding capacity (g/g)		MIU	8.45^Aa^	8.36^Aa^	7.24^Aa^	7.85^Aa^	7.37^Aa^	7.48^Aa^
		OEM	5.13^ABb^	5.07^ABb^	5.24^ABb^	4.85^ABb^	6.14^Ab^	4.37^Bb^
density (g/mL)		MIU	0.673^Ab^	0.609^Ab^	0.618^Ab^	0.636^Ab^	0.603^Ab^	0.618^Ab^
		OEM	0.934^Aa^	0.932^Aa^	0.906^Aa^	0.923^Aa^	0.931^Aa^	0.933^Aa^
granulometry (mm)	ASTM 35	MIU	1.47^Ca^	2.08^Aa^	1.72^Ba^	1.03^Ea^	1.03^Ea^	1.17^Da^
		OEM	0.97^Bb^	1.38^Ab^	0.84^Db^	0.81^Db^	0.67^Eb^	0.92^Cb^
	ASTM 60	MIU	3.14^Db^	2.61^Fb^	2.98^Eb^	3.50^Ba^	3.60^Aa^	3.35^Ca^
		OEM	3.23^BCa^	2.93^Da^	3.36^Aa^	3.18^BCb^	3.25^Bb^	3.15^Cb^
	ASTM 270	MIU	0.24^Bb^	0.16^Cb^	0.18^Cb^	0.30^Bb^	0.28^Bb^	0.44^Ab^
		OEM	0.57^Da^	0.48^Ea^	0.67^Ca^	0.83^Ba^	0.96^Aa^	0.85^Ba^

a ASTM 35, 60, and 270 correspond to diameters 0.5, 0.250, and 0.053 mm, respectively.

b Values with different letters between columns show a significant difference (*P* < 0.05). Uppercase letters denote time (months), and lowercase letters denote plant model species.

With respect to the granulometry of the mucilage of the species studied, in the 0.50 mm mesh, it was observed that there was a decrease in the number of particles over storage time (Table 1). Differently, in the two other meshes (0.250 and 0.053 mm), the number of particles increased over the experimental time. Also, the ASTM 60 mesh (0.250 mm) retained more than 60% of the mucilaginous grains in both species (Table 1).

OEM or MIU mucilage showed stability in terms of water and oil retention capacities up to 8 months of storage (Table 1). In addition, regardless of storage time, the MIU species showed higher average values of water and oil retention capacities (Table 1).

The transparency of the films of both species increased as time passed (Table 1). On the other hand, the thickness decreased up to the eighth month for OEM, while MIU thickness continued to reduce until the end of the study (Table 1). Furthermore, the films resulting from MIU mucilage were significantly thicker at the end of the storage time (Table 1).

**Figure 1:**
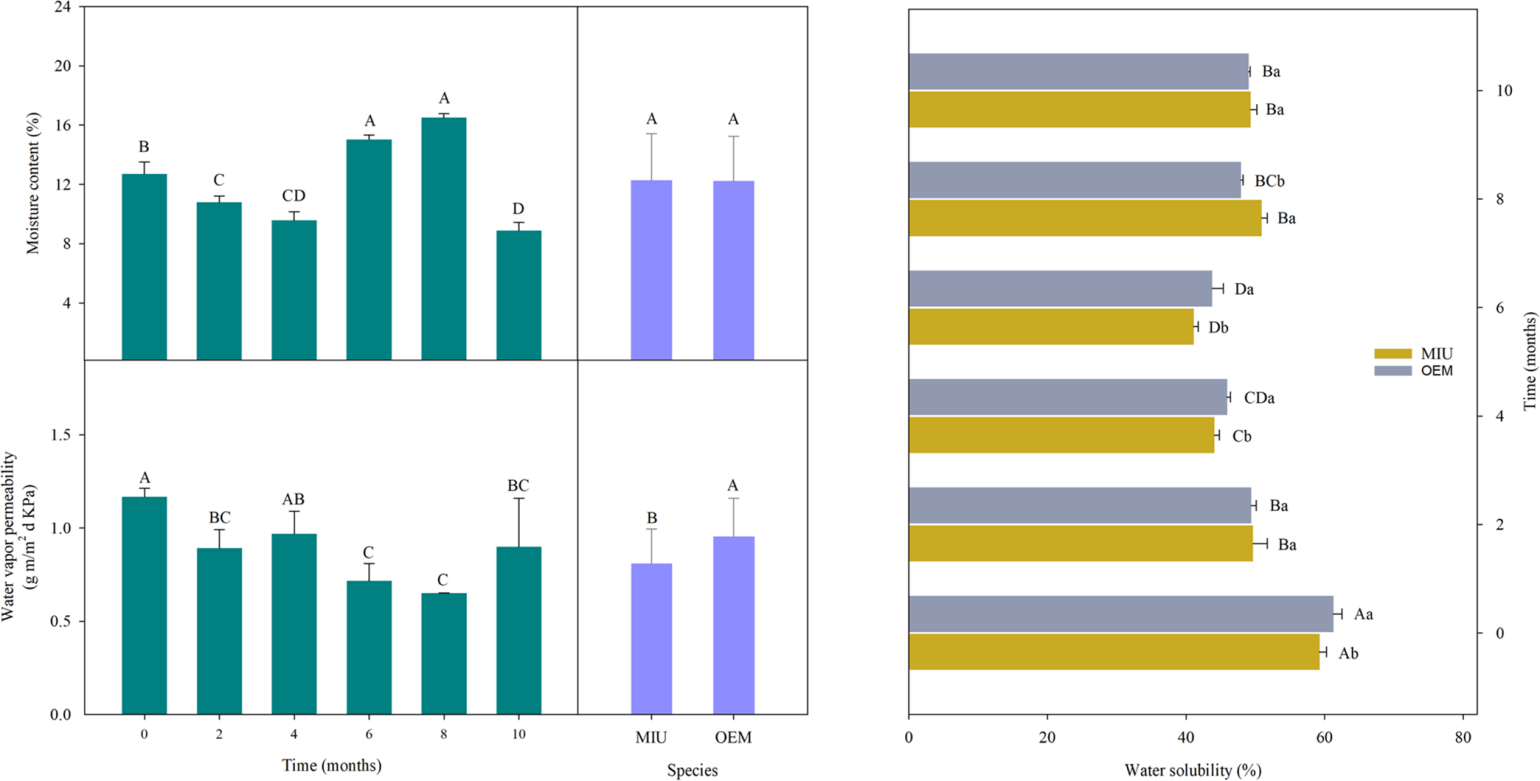
Functional properties of polymeric films obtained with the mucilage *N. cochenillifera* (L.) Salm-Dyck MIU, and *O. stricta* (Haw.) Haw—OEM, immediately after the cladode harvest and after 2, 4, 6, 8, and 10 months. The bars represent the standard deviation of the mean. Values with different letters show a significant difference (*P* < 0.05).

The moisture content of the films was at its highest at 8 months and lowest in the 10th month, regardless of the species (Figure 1). On the other hand, permeability decreased, reaching minimum values at 6 and 8 months for both species (Figure 1). It was also noted that the average permeability was higher for the OEM species (Figure 1). Water solubility also followed the reduction in water vapor permeability (Figure 1).

### Mechanical and Thermal Properties

Mucilage maintained satisfactory overall results for 8 months. The tensile strength of the films based on cactus pear mucilage showed a gradual reduction in values, with MIU having significantly higher values (Table 1). The OEM species showed an increase in the first months, but after the eighth month, the values decreased (Table 1).

**Figure 2:**
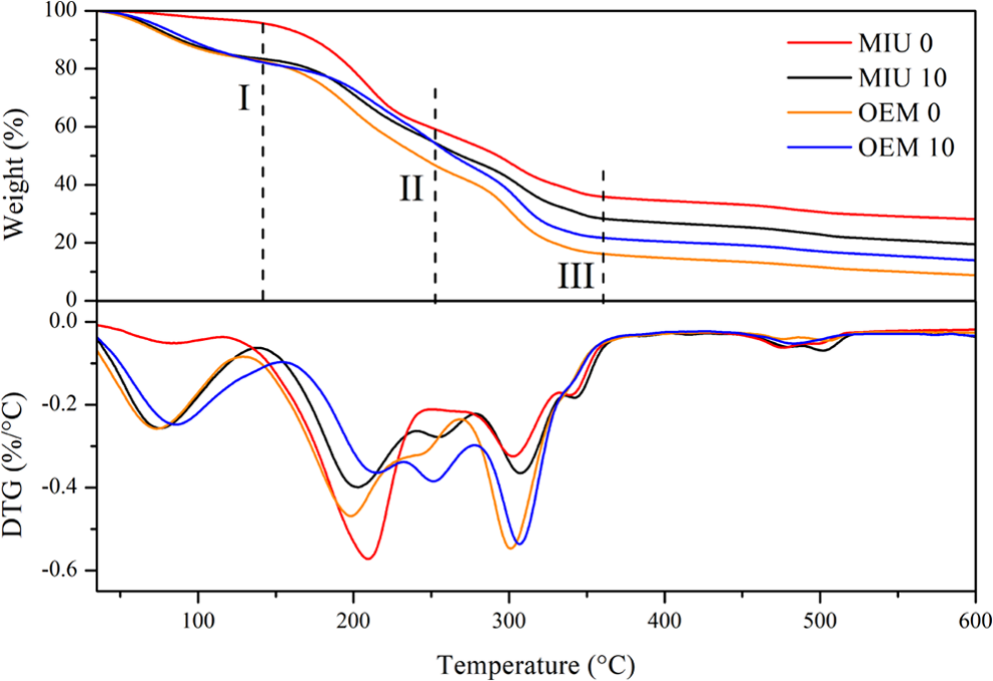
Thermal stability of polymeric films based on cactus pear mucilage *N. cochenillifera* (L.) Salm-Dyck—MIU and *O. stricta* (Haw.) Haw—OEM, immediately after cladode harvesting (0) and after the storage of 10 months (10). The derivative thermogravimetry (DTG) peaks represent the degradation temperature at each stage.

MIU films had less mass loss, and their degradation occurred at temperatures higher than OEM in all degradation stages (Figure 2). When subjected to heating, dehydration occurs due to the removal of water molecules, fragmentation, and degradation of the polymer matrix.^18^

In the films studied, thermogravimetry was evaluated at the beginning and end of the experiment. The weight loss of the samples occurred in three stages due to the complexities of the polysaccharide matrix of the mucilage. The first stage showed 25% mass loss of initial and final OEM and final MIU, while initial MIU showed 0.05%; at 73 and 86 °C, respectively. This refers to the loss of moisture from the films by evaporation. The second stage presented losses of 40–60% between 200 and 210 °C, for MIU and OEM, respectively, related to the degradation of the mucilage side chains. The third and last stage showed losses of 60% of the mass of the OEM polymer in the range of 300 °C, while MIU presented losses of 35%; these losses are related to the main chain of mucilage monosaccharides in the range between 250 and 350 °C where dehydration of the monosaccharide rings and depolymerization occurs.^19,20^

### Optical Properties

The luminosity increased significantly up to 8 months regardless of the species (Table 1). Chroma values progressively increased over time for MIU, unlike OEM, which showed a decrease in the values during storage (Table 1).

**Figure 3:**
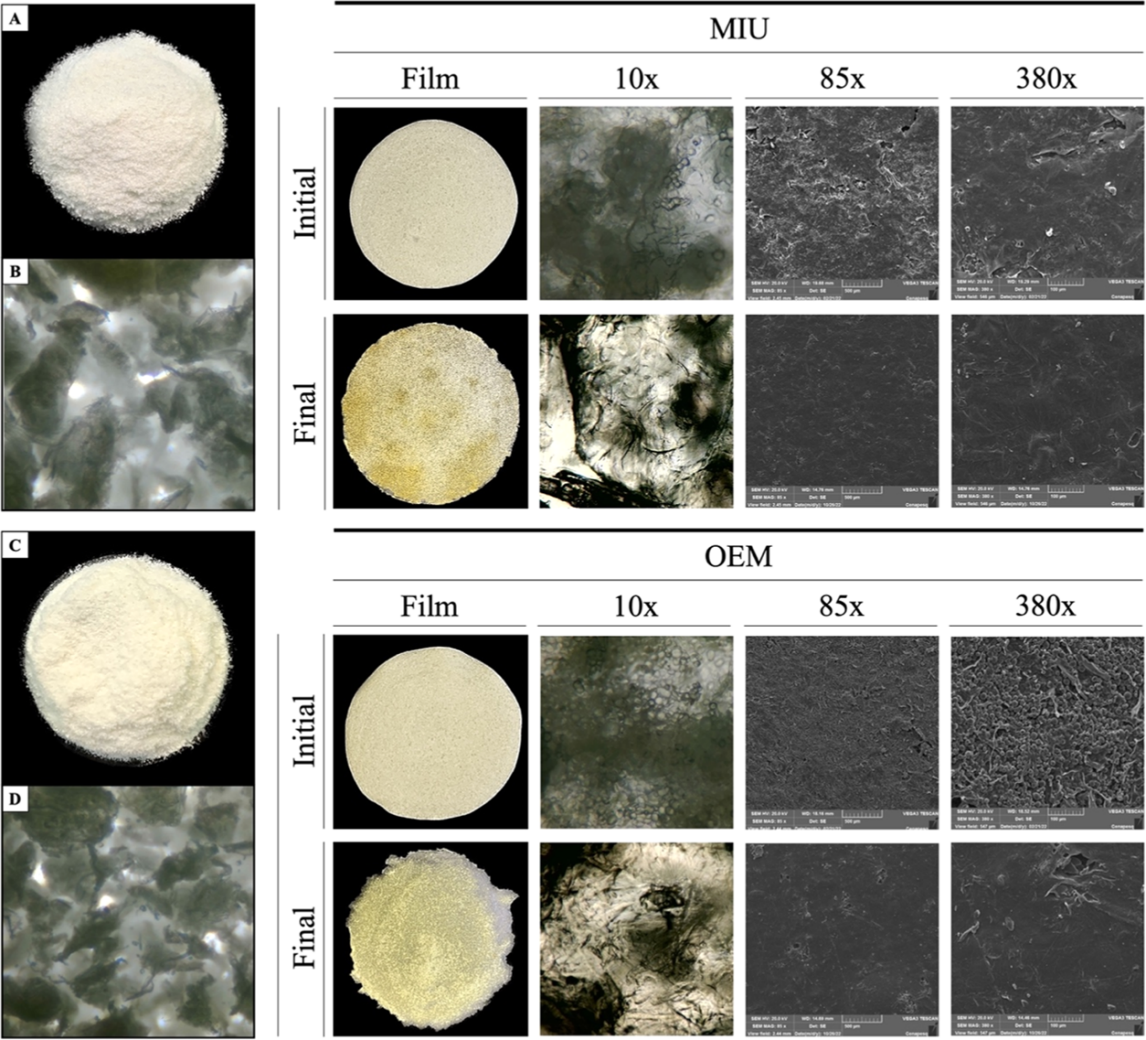
Visual appearance and micrographs of the mucilage and polymeric films from cactus pear mucilage. Macro images in (A) and (C), zoomed in at 10× in (B) and (D). In the films, macro imagens and zoomed in at 10×, 85×, and 380×. Initial corresponds to the beginning, and final, after 10 months. *N. cochenillifera* (L.) Salm-Dyck MIU and *O. stricta* (Haw.) Haw—OEM.

The mucilage extracted from the MIU species showed a whitish color compared to that of the OEM species, which showed a slightly yellowish hue (Figure 3A,C). In the photomicrographs, OEM appears to contain slightly smaller particles compared to MIU (Figure 3B,D).

The biopolymeric films resulting from MIU presented a whitish color compared to those from OEM, both at the beginning and at the end of the study (Figure 3). Magnified SME images show that the MIU films are more compact and homogeneous; unlike what can be observed in the OEM polymeric base, which showed more dispersed particles in its matrix (Figure 3). These results were repeated at the end of the study, but the biopolymeric films of both species became more homogeneous and denser.

### Fourier Transform Infrared (FTIR) Spectrophotometry and Principal Component Analysis (PCA)

**Figure 4:**
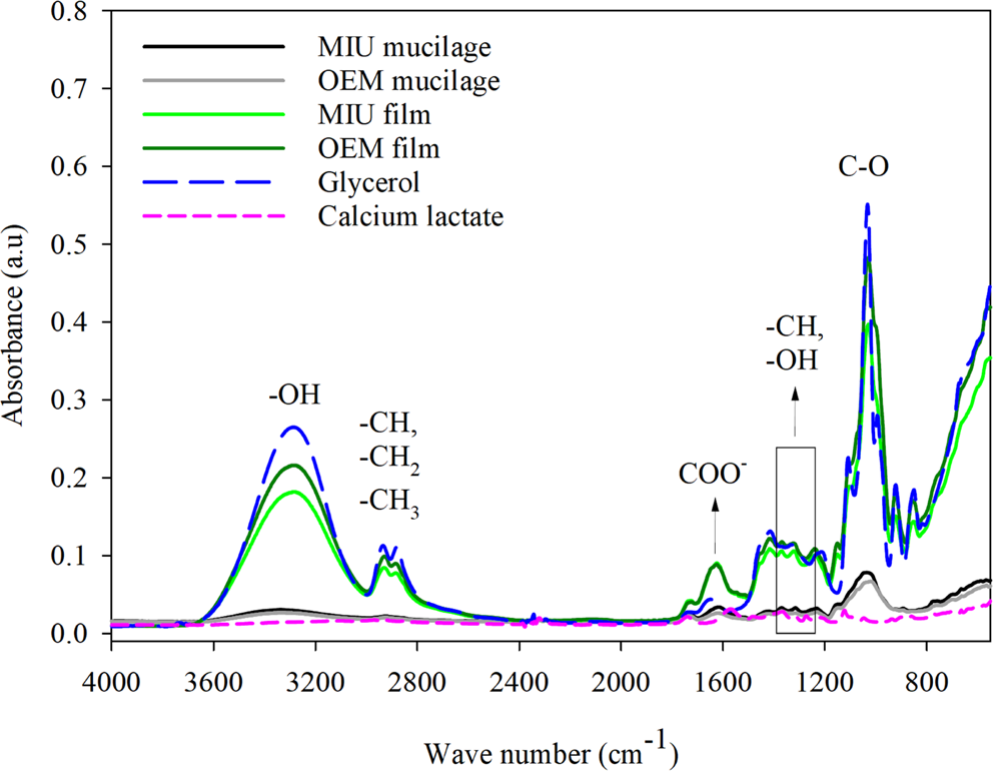
Infrared spectrum with mean curves of MIU (*N. cochenillifera* (L.) Salm-Dyck) and OEM (*O. stricta* (Haw)). Haw mucilage and their resulting films, immediately after cladode harvesting and after 2, 4, 6, 8, and 10 months, and comparison with the spectra of the plasticizer used (glycerol) and the matrix additive (calcium lactate).

The spectral behavior in the infrared (FTIR) of MIU and OEM mucilages at the beginning of the experiment and throughout storage was similar for both. This behavior was also common to films formulated over time and in their respective species. Thus, the average curves of the treatments studied in storage were used and the spectra of the components present in the formulation of biopolymeric films (glycerol and calcium lactate) were added (Figure 4).

**Figure 5:**
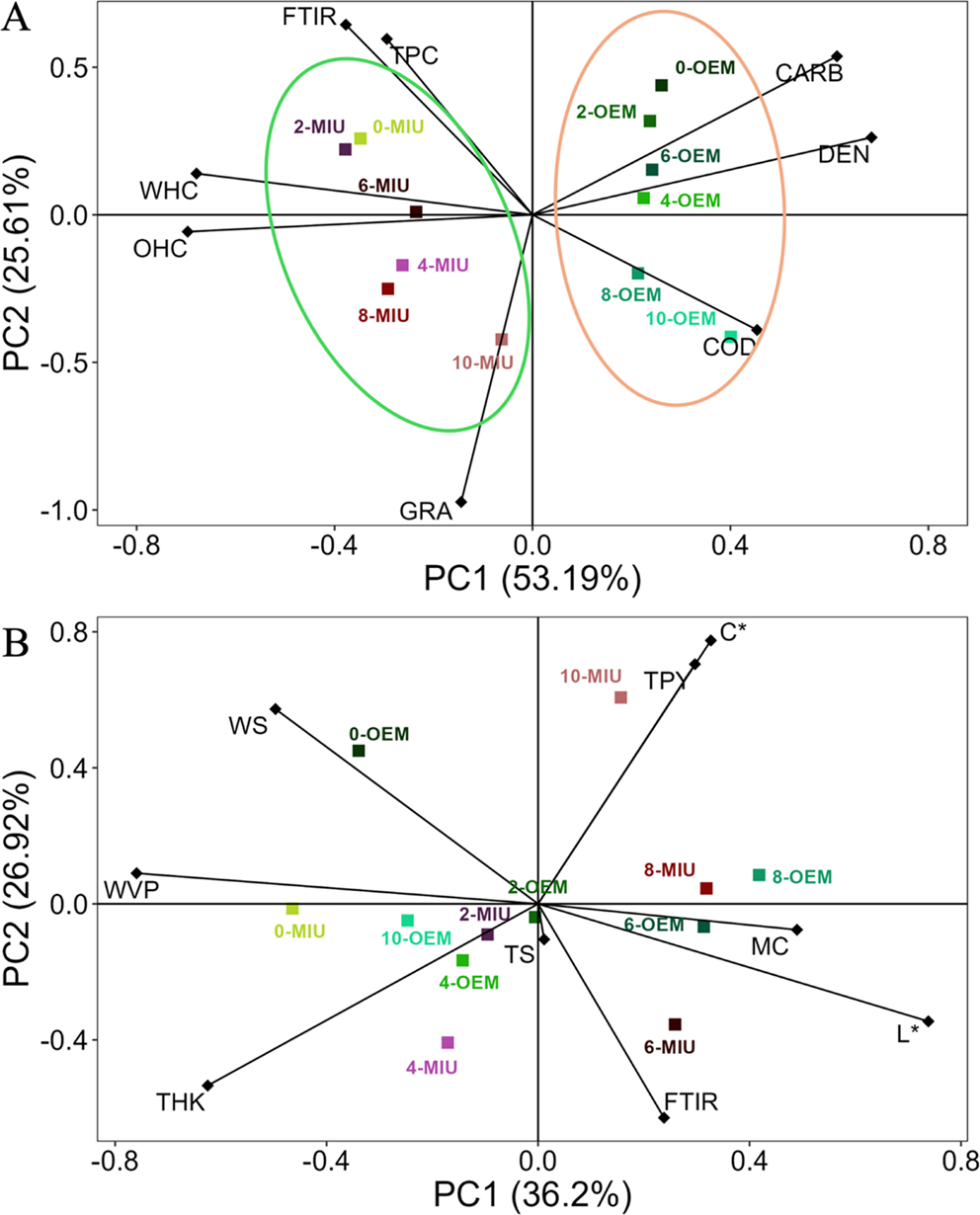
Biplots of principal component analysis based on standardized means of mucilage variables (A) and films (B) immediately after cladode harvesting and after 2, 4, 6, 8, and 10 months for the studied species, *N. cochenillifera* (L.) Salm-Dyck MIU and *O. stricta* (Haw.) Haw—OEM. Note: WS: water solubility; TPC: total phenolic compounds; FTIR: infrared spectroscopy; WHC: water holding capacity; OHC: oil holding capacity; GRA: granulometry; COD: conductivity; DEN: density; CARB: total soluble carbohydrates; WVP: water vapor permeability; THK: thickness; MV: maximum voltage; FTIR: infrared spectroscopy; TS: tensile strength; L*: brightness; MC: moisture content; C*: chroma; TPY: transparency.

The PCA of the mucilage was explained by 78.8%, a total variation composed of two main components, the PC1 with 53.19%. Greater contributions were from the following variables: total phenolic compounds (TPC), infrared spectrophotometry (FTIR), water holding capacity (WHC), and oil holding capacity (OHC). These were negatively correlated with total soluble carbohydrates (CARB), density (DEN), and conductivity (COD). Lower values of CARB, DEN, and COD were observed in MIU samples, and higher values of TPC, FTIR, WHC, and OHC. The second principal component (PC2) accounted for 25.61% of the data variance, mainly due to granulometry (GRA) which are negatively correlated (Figure 5A).

The variation in the PCA of the films was 63.12% due to two main components. These variations were composed of two main components: the PC1 with 36.2% of the variables including the following: infrared spectrophotometry (FTIR), luminosity (L*), moisture content (MC), transparency (TPY), chroma (C*) and tensile strength (TS); and negatively correlated with water solubility (WS), water vapor permeability (WVP) and thickness (THK). Higher values of WS, WVP, and THK were observed in the MIU samples at 0, 2, and 4 months and OEM at 0, 4, and 10 months; higher values of TPY, C*, MC, L*, FTIR, and TS were found at 6, 8, and 10 months of storage for MIU mucilage and at 2, 6, and 8 months for OEM mucilage. The second principal component (PC2) was responsible for 26.92% of the data variance (Figure 5B).

## Discussion

The manufacture of biopolymeric films and coatings using cactus mucilage as a matrix has grown in recent years.^21^ However, it is observed that the mucilage obtained for the manufacturing of biopolymeric films originates from freshly harvested cladodes.^22,23^ There is a gap regarding the actual possible storage time of cactus pear mucilage for use in food or in the production of biopolymeric films. Therefore, it was studied two plant models of cactus pear, the species *N. cochenillifera* (L.) Salm-Dyck—MIU and *O. stricta* (Haw.) Haw—OEM, the latter being the most cited in the literature.^23,24^ In Brazil, we initiated studies with Nopalea species, which showed potential for use in the production of biopolymeric films.^14,22^

The raw material of the study was the result of the bioprospecting of cladodes of MIU and OEM. The great challenge in obtaining mucilage is related to yield, which, when low, represents disadvantages for the industry due to the economic unviability of its production. In the present study, the yield of both species was on average 0.95% based on fresh mass, a result lower than that already found by the group and presented in the article by Costa de Sousa and collaborators,^4^ which presented about 10% yield in obtaining the mucilage of MIU. The results of the present study are similar to those obtained by Dick et al.,^25^ which presented 1.20%. Further, in our study, the powder resulting from the extraction of MIU was more whitened in relation to that from OEM (Figure 3A,C), possibly due to the higher concentration of pigments in the OEM in relation to MIU. The reflection in the change in mucilage color was observed in biopolymeric films, where the MIU was more whitish (Figure 3), confirmed by the higher luminosity values in relation to OEM (Table 1), these values are similar to those seen *Opuntia ficus-indica* (69–99 L*)^26^ and are high values, but then, the scale has a maximum value of 100 L*; on the other hand, lower chroma values were seen for MIU (Table 1). These confirm higher OEM saturation, with yellowish-green color linked to the expressiveness of these results.^15^ These results are supported by the C* values that were found. It is reported that the color changes in biopolymer films are due to anthocyanin extracts, compounds influenced by pH.^27^ In addition, there was a difference in the homogeneity of the films, and MIU was more homogeneous than OEM. Photomicrographs observed the surface by scanning electron microscopy (SEM). Nonhomogeneous points were observed in MIU polymer at an 85× zoom. These points or pores can facilitate the minimum necessary gas exchange (1–3% oxygen), preventing their entering anaerobic and fermentative routes when incorporated into food.^28^ However, at a zoom of 380×, the surface of MIU was observed to be more homogeneous in relation to OEM, where clusters were perceptible, directly interfering with other properties of films and their flexibility. At a film storage time of 10 months, the photographs show a clear reduction in the visual quality of the films. These changes were seen in the photomicrographs by SEM, presenting gelatinous and homogeneous aspects indicative of a gradual reduction in the properties of mucilage.

Mucilage contains important physical–chemical parameters that makes it adaptable to the most diverse uses.^3^ The acidity did not change according to species or with the storage of mucilage (Table 1). On the other hand, soluble carbohydrates and total phenolic compounds fell significantly over the months, regardless of the species studied (Table 1). Mucilage is a complex carbohydrate^6^ with a seldom explored polysaccharide structure, although this has an influence on the filmogenic properties of mucilage. The increase in phytochemicals such as phenolic compounds has a negative effect on filmogenic properties because their reaction through ester bonds with polysaccharides such as galactose and arabinose can increase water barrier properties and reduce their filmogenic potential.^14,29^ In the films formulated, there was a variation over the months for OEM and MIU, but the latter presented higher resistance (Table 1) and thermal stability (Figure 2), suggesting that MIU films may be suitable for applications such as biopolymeric films and edible coatings from powdered mucilage stored for up to 8 months, a common practice in industry.^3^ Transparency increased over the months, but MIU presented lower values and, proportionally, there was a reduction in the thickness of the films, with emphasis on the better results in MIU. Less transparency is desired to avoid exposure to UV rays to prevent oxidative damage as a barrier against the external environment.^30^ In addition, thicker biopolymeric films or films are reported to be durable because they may last longer when applied to other surfaces.^14^ MIU also showed significantly reduced water vapor permeability of its films which decreased over the months, its results were lower than the other reported ranges, between 1.27 and 5.29 g m/m^2^ s kPa.^17^ Due to the greater homogeneity, MIU films guarantee less exposure of the product to the environment, which is a desirable parameter for the food industry.^31^ Moisture content varied over storage time without distinction between the species, but remained similar to other reported values, between 8.2 and 15% (Figure 1).^29^ Solubility in water was different (Figure 1), presenting much higher results compared to those reported by Sukhija et al. whose highest treatment value was 28%.^17^

Electrical conductivity, highly related to viscosity, increased significantly over storage time in the studied species: the increase in MIU was 39.5%, while the OEM increased 20.5% (Table 1). Viscosity interferes in consequent applications of mucilage for polymeric formulations, as it is influenced by the concentration of monovalent and divalent ions present in the mucilage.^7^ With the gradual detachment of ions, there is a reduction in viscosity due to molecular disarrangements^6^ caused by negative charges in systems without a counterion, causing intermolecular repulsion and expansion or swelling of molecules.^32^ The solubilities of the films were similar in both species. It is reported that the increase in temperature triggers increased swelling and solubility^33^ and may be related to the destruction of weak intermolecular forces of mucilage molecules, causing increased water trapping by molecules. The mucilage was stable in terms of water retention capacity (WHC) and oil (OHC) during the study, but MIU showed higher values. The results presented for MIU and OEM are higher than those reported for *O*. *ficus-indica*, which presented 7.81 g/g of WHC and 1.34 g/g of OHC,^34^ related to the affinity of the greater mucilage water retention, as already reported in the literature,^8^ explaining the reduced rate of oil retention. These results may also be associated with the hydroxyl groups present in mucilage,^35^ the presence of carbohydrates, and other functional groups that favor the interaction between mucilage and water.^34^ However, in the last storage month, there was a significant decrease in the results in the mucilage for both species regarding WHC, indicative of the loss of quality of the material due to time. Another physical property of mucilage determined was density, which was stable after months of storage, but OEM presented higher values (Table 1). MIU and OEM mucilage particles did not vary in size in the meshes with higher concentrations (0.250 mm), where they gathered 63% of their mass, in agreement with the reported range of 200–500 mm.^36^ Ground and sieved particles can improve properties in addition to their organoleptic quality and provide food stability.^36^

The general profile of the spectra of the mucilage samples and the films submitted to time were similar, so the mean curve of the months studied in the different species was used, which contained results similar to those found in the literature.^1,5^ The bands between 3400 and 3200 cm^–1^ are related to O–H vibrations of alcohol and carboxylic acid (−C(O)–OH) groups correlated with hydrogen bridges and OH bonds between molecules, characteristics common in water molecules.^14^ The peaks observed at 2932 and 2888 cm^–1^ were designated as elongation C–H, CH_2_, and CH_3_ and traces of carboxylic acid and aldehyde.^1^ In addition, band 1612 cm^–1^ was assigned to the COO (carboxylate ion).^1^ A set of bands with absorbents between 1400 and 1240 cm^–1^ can be assigned to groups C–H or −OH.^37^ In addition, higher intensity was observed in the band around 1047 cm^–1^, observed as C–O stretch corresponding to alcohols, carboxylic acids, esters, and ethers.^3^ Peaks resulting from vibrations below 1200 cm^–1^ are related to the presence of carbohydrates in mucilage, but cannot be identified specifically due to their complexity.^14^ It is possible to visualize a similar spectrum in films with glycerol, which demonstrates the influence of glycerol on the polymeric films. Calcium lactate is seen as a curve with low peaks compared with the other curves observed.

The present study showed the influence of storage on the different properties observed in mucilage and films of the species under research, MIU and OEM. An analysis of the main components (PCA) of mucilage indicates a tendency for group formation among the studied species (Figure 5A) intensifying the changes between the properties of the two species. The grouping of a higher number of physical–chemical variables in the OEM mucilage reinforces the mucilage stability of this species. However, MIU mucilage had higher water and oil capacities (Table 1), and higher peaks in the infrared spectroscopy (Figure 4). This may suggest that the technological properties of this species are more pronounced and, therefore, more useful in the formation of polymeric bases. In the films, PCA also presents a tendency to form groups by month and by species (Figure 5B), intensifying the differences among the properties. In the initial months, MIU presented better results for important variables in the characteristics of films such as resistance, thickness, and permeability, due to its decay over time, and showing at 6, 8, and 10 months changes in color values (L* and C*), transparency, moisture content, and FTIR. On the other hand, OEM did not present this cohesion in the results over time, having some months with higher values than others, which shows a certain instability of the material in the formulation of films. In line with the results observed for mucilage, the study of the films suggests that MIU mucilage has a strong potential for the formulation of films for various applications in the industry.

## Conclusions

The study of storage over time of MIU and OEM mucilage and their films indicated that storage was successful and applicable for 8 months and thus adequate for potential industrial demand. Among the two species, MIU stood out due to its higher water and oil retention capacity as well as exhibiting higher levels of phenolic compounds, swellability, and more intense peaks in FTIR analysis. On the other hand, OEM is richer in carbohydrates, has greater density, and is more electrically conductive. Both species are equally soluble in water, and more than 60% of their granules have a diameter of 250 mm. The distinctive characteristics of each species are also evidenced in the principal component analysis (PCA). Additionally, films produced with MIU showed greater strength, thickness, and water vapor permeability along with lower values of transparency and solubility, which are important parameters in biopolymeric films intended for application in food. However, the study supports both species as potential candidates for film production.
